# Factors related to complications of the invasive blood pressure system among adult and elderly patients: a prospective study

**DOI:** 10.1590/1518-8345.7097.4443

**Published:** 2025-01-31

**Authors:** Willian Marques de Souza, Victor Hugo Baldan Cypriano, Rosângela Aparecida de Sousa, Rafael Luis Bressani Lino, Ana Luiza Mroczinski, Danielle Cristina Garbuio

**Affiliations:** 1 Centro Universitário Central Paulista, Departamento de Enfermagem, São Carlos, SP, Brazil.; 2 Universidade Federal de São Carlos, Departamento de Enfermagem, São Carlos, SP, Brazil.; 3 Universidade de São Paulo, Escola de Enfermagem de Ribeirão Preto, PAHO/WHO Collaborating Centre for Nursing Research Development, Ribeirão Preto, SP, Brazil.

**Keywords:** Nursing, Hemodynamic Monitoring, Arterial Pressure, Nursing Care, Advanced Practice Nursing, Critical Care Nursing

## Abstract

**Objective:**

to identify the main factors related to complications of the invasive blood pressure system.

**Method:**

prospective study conducted with patients over 18 years of age admitted to intensive care, using a device for measuring invasive blood pressure. Participants were monitored during the catheter dwell-time and sociodemographic, clinical and device data were collected. The outcome analyzed was removal due to non-indication of use or due to complications. Student’s t-test, Mann-Whitney U test, chi-square test and Fisher’s exact test were used for the analyses.

**Results:**

50 participants were included and monitored, and most devices were installed in the radial artery (86%), with a 20-*gauge* catheter (50%), all with a flexible catheter. Each patient remained, on average, 4.36 days (SD: 3.504) with the device. Regarding the outcomes, 60.0% of the devices were removed due to non-indication of use and 40.0% due to complications. Phlebitis was the most prevalent complication, and pressure in the bag was the factor associated with catheter removal before the time of indication (p=0.046).

**Conclusion:**

the main complications associated with this device were obstruction and phlebitis, while pressure in the bag was the factor related to catheter removal before indication.

## Highlights

**(1)** Phlebitis was the most prevalent complication.

**(2)** Pressure in the bag was associated with catheter removal before indication.

**(3)** Length of stay and use of sedation were related to the onset of phlebitis.

## Introduction

Arterial cannulation is a common procedure in acute and critical care settings. It refers to an invasive method for accurately measuring blood pressure and mean arterial pressure, thus providing measurements that allow immediate recognition of changes and rapid intervention. In addition, it offers less traumatic access to frequent arterial blood collection^(1-4)^.

This procedure is recommended for critically ill patients with hemodynamic instability, such as hypertensive emergencies, shock states, use of vasoactive amines, vasodilators, vasopressors and inotropes, as well as for patients in the immediate postoperative period for cardiac and neurological surgery. It is also suitable for situations in which the patient cannot undergo noninvasive blood pressure monitoring, in cases of burns covering a large area of ​​the body or multiple extremity fractures^(4-5)^.

For properly measuring and analyzing invasive blood pressure values (IBP), the catheter must be inserted, positioned and maintained properly. Initially, the puncture site and the gauge size of the catheter to be inserted are chosen and, after puncture is performed, it is essential that the transducer is leveled to the phlebostatic axis, ending with a critical observation of the blood pressure wave on the multiparameter monitor^(3)^.

The recommended puncture sites for monitoring IBP or collecting arterial blood are the radial, dorsalis pedis, and femoral arteries. Due to easy access and low risk of contamination and loss, the radial artery is the preferred site for puncture and insertion of the device^(1,6)^. On the other hand, in the case of patients in refractory shock, monitoring IBP through the radial artery produces less accurate pressure values when compared to femoral puncture^(7-8)^. The type of catheter and its gauge size will depend on several factors, such as the artery to be punctured and the risks involved in the procedure^(3)^.

For radial artery cannulation, the Allen Test is recommended, which aims to evaluate the collateral circulation of the ulnar artery in order to avoid ischemic injury^(1)^. However, studies show that it has low diagnostic accuracy, and therefore should not be considered a predictor of ischemic complications related to radial artery puncture^(3)^.

Absolute contraindications to the insertion of an arterial catheter include absence of pulse, Raynaud’s syndrome, inadequate or interrupted circulation, and infection at the insertion site. Cases of coagulation disorders and anticoagulation situations, burns, arterial grafts, and surgical interventions at the insertion site must be individually assessed^(1,4)^.

In terms of complications related to arterial puncture for invasive monitoring purposes, they will depend on the insertion site^(2,9)^ and, in general, pain at the site is observed, as well as paresthesia, hematoma, bleeding, ischemic complications, embolism, vascular thrombosis, occlusion, vascular injury, pseudoaneurysm formation, abscess and local nerve injury^(1-3,9)^. Serious complications related to this procedure are not frequent, but a risk-benefit analysis must be performed for each patient^(4)^. Furthermore, recent studies show that ultrasound-guided arterial puncture is significantly related to the reduction of failures in the procedure^(10)^.

In Brazil, within the nursing team, arterial puncture, both for collecting arterial blood gas and for hemodynamic monitoring purposes, is a practice exclusive to nurses. Therefore, it is essential that these professionals be trained and qualified to install the IBP device, which includes the possibility of using bedside ultrasound to assist with the puncture and manage the anesthesia pump to secure the catheter^(11)^.

In this context, nurses must have knowledge on how to insert IBP devices as well as be aware of the main complications of this procedure and possible predisposing factors with the purpose of providing qualified nursing care and developing targeted care plans^(12)^. In view of the above, the following guiding question was developed for this study: What are the main factors related to complications of the IBP system among adult patients? To answer this question, it was defined that the objective was to identify the main factors related to complications of the IBP monitoring system.

## Method

### Study design

Quantitative, observational, prospective study, guided by the Strengthening the Reporting of Observational Studies in Epidemiology (STROBE)^(13)^.

### Location and period of collection

Data collection was carried out in July and August 2022 at a philanthropic hospital located in the interior of the state of São Paulo. The hospital has three adult intensive care units, one specifically for cardiology with 15 beds, and two for general care, each with 10 beds. The study was developed in these three units.

### Population and selection criteria

Patients over 18 years of age (adults and elderly) admitted to the aforementioned intensive care units, with an indication for use of an invasive blood pressure measuring device (patients in shock, using vasoactive drugs, hemodynamically unstable, who underwent major surgery, frequent blood gas collection, using inotropic drugs, who had hypertensive crises, among others)^(3)^ installed in the aforementioned units in the last 48 hours were included. The level of consciousness was not an eligibility criterion, since, due to the health status of the participants, many could be sedated or have an impaired level of consciousness. Therefore, it was decided to forward the call for participation to the patients’ family members.

### Data collection and study variables

Data collection was carried out through daily visits to the units in order to search eligible participants and update data on those being monitored. When the patient met the inclusion criteria, the family member was contacted and informed about the research, its objectives, and risks and, if accepted, they were requested to sign the Free and Informed Consent Term (Portuguese acronym: TCLE) to register their consent.

Once accepted, sociodemographic data including age, gender, race, smoking status, alcohol use, comorbidities, eating habits, Body Mass Index (BMI), devices and medications in use were collected from the medical records. The following data related to the IBP were collected: puncture sites, catheter gauge size and type, volume of saline solution and type of bandage used, and pressure in the bag. On subsequent days, the type of bandage, the level of pressure in the pressure bag, catheter permeability and signs of phlebitis (using the visual phlebitis scale of the Infusion Nursing Society^(14)^) were assessed. When the catheter was removed, either by indication or due to complications, participation in the study was terminated.

The predictor variables assessed were the following: age, gender, race, smoking status, alcohol use, comorbidities, eating habits, BMI, devices and medications in use, type of bandage, puncture sites, catheter gauge size and type, volume of saline solution and type of bandage and level of pressure in the bag. In turn, the outcomes evaluated referred to the removal of this device before the scheduled time due to infection or obstruction, death or even because its use was not indicated.

Participants were monitored daily until the device was removed by indication, in case of clinical improvement, due to complications or death. Therefore, scheduled removal was considered as the one performed according to a prior planning and removal due to complications as the one performed due to infection, obstruction or accidental removal. For analytical purposes, removal due to death was considered as removal by indication.

### Data treatment and analysis

The collected data were entered into Microsoft Excel^®^ spreadsheets with double entry. The variables were initially analyzed using descriptive statistics for each variable and their distributions were verified using the Shapiro-Wilk test; subsequently, the incidence of complications was determined. To calculate the incidence, the total number of removals due to complications was divided by the total number of punctures. Continuous variables that followed a normal distribution were compared for the groups with and without complications using the Student’s t-test. The effect size of the difference between the means was assessed using Hedges’ g, considering the reference values for the effect size^(15)^ as insignificant for values lower than 0.19 and medium for values between 0.50 and 0.79. The continuous variable that failed to follow a normal distribution was assessed between the groups using the Mann-Whitney U test and, in this case, the effect size was calculated using Pearson’s r^(16)^. For categorical variables, the correlation with the outcome was performed using Pearson’s chi-square test or Fisher’s Exact test (if the values shown in the columns were lower than 5). The relative risk and respective confidence intervals were also calculated for these analyses. The IBM SPSS Statistics 22^®^ software was used considering a significance level (α) of 5%.

### Ethical aspects

The research project was submitted to the Human Research Ethics Committee for consideration and approved under report No. 5.484.178 of 2022. Companions and participants were approached ethically in order to be informed on the purposes of the research and to state their respective consent by signing the Free and Informed Consent Term (Portuguese Acronym: TCLE). All ethical precepts determined by Resolution No. 466/12 of the National Health Council of the Ministry of Health were duly followed.

## Results

**Figure 1:**
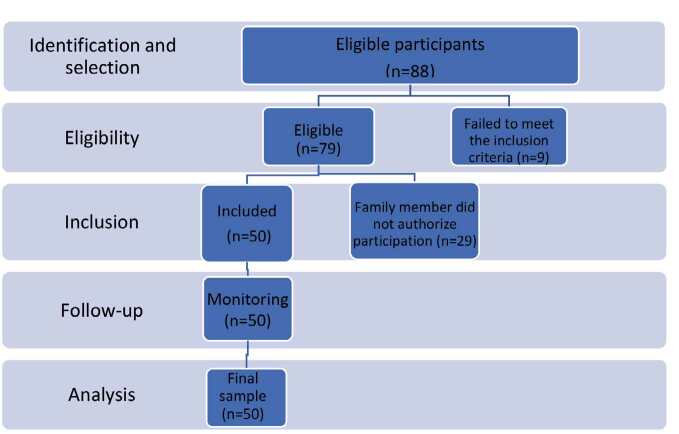
- Screening, inclusion and follow-up flowchart of the research participants. São Carlos, SP, Brazil, 2022

During the data collection period, 88 eligible participants were selected for the research. The screening and inclusion flowchart is shown in Figure 1.

The average age of the participants was 58.1 years (SD: 16,337), most were men (56%), white (60%), and single (48%). Table 1 shows the sociodemographic and clinical characterization of the participants.

**Table 1:** - Sociodemographic and clinical characterization of the participants and information on their invasive blood pressure system (n = 50). São Carlos, SP, Brazil, 2022

	**N**	**%**
**Sex** Male Female	28 22	56 44
**Race/Skin color** White Brown Black	30 17 03	60 34 03
**Smoking status** Smoker Non-smoker Former smoker Not identified	08 21 14 07	16 42 28 14
**Alcohol use** Alcohol user Does not drink alcohol Former drinker Not identified	16 19 04 11	32 38 08 22
Comorbidities* Diabetes Mellitus Systemic Arterial Hypertension Chronic Kidney Disease Heart diseases Others	16 36 00 02 28	32 72 00 04 56
**Eating habits** Oral route Nasoenteric tube	21 29	42 58
**Vasoactive drug** Yes No	48 02	96 04
**Sedation**Yes No	33 17	66 34
**Antibiotic** Yes No	37 13	74 26
**Anticoagulant** Yes No	18 32	36 64
**Central venous catheter** Yes No	37 13	74 26
**Puncture site** Radial Dorsalis Pedis	43 07	86 14
**Volume of pressurizing solution** 250 ml 500 ml 1000 ml	1 13 36	02 26 72
**Bandage used at the time of puncture** Transparent and Waterproof Gauze and Micropore	32 18	64 36
**Reasons for removal** Non-indication of use/scheduled Complication Death	24 20 06	48 40 12

*Each patient could have more than one comorbidity, with the sum exceeding 50

Considering the characteristics of the invasive blood pressure measuring devices, most were installed in the radial artery (86%), all using a flexible needleless catheter. During the period established for collection, no device was installed in the femoral artery. It is noteworthy that the Allen test was performed in 86% of the radial punctures. The most used catheter was the 20-*gauge* (G) (50%), followed by the 18G (42%) and, lastly, by the 22G (8%).

Each patient remained, on average, 4.36 days (SD: 3.504) with the device, for a minimum of 1 day and a maximum of 16. The incidence of complications reached 40% and, among them, 52% of the punctures showed signs of phlebitis, 36.54% signs of obstruction and 11.46% showed other complications. In all cases of complications, the catheter was removed and the study was not continued after the new puncture.

The variables age (p=0.156), BMI (p=0.725) and mean pressure in the bag (p=0.153) showed normal distribution assessed by the Shapiro-Wilk test. In turn, the variable “catheter dwell time”, measured in days, was assessed by the same test and failed to present normal distribution (p<0.000).

In this study, no relationship was found between the outcome (removal due to complications or by indication) and the variables gender, age, dwell-time, use of anticoagulants, antibiotics or sedation, eating habits, catheter gauge size and other factors, as shown in Tables 2 and 3.

**Table 2:** - Comparison between the outcomes and mean values for age, device usage time and pressure in the pressurizing bag (n = 50). São Carlos, SP, Brazil, 2022

**Variable**	**Complication**	**Mean**	SD*	**p-value** ^†^	**Hedges’g**	**Effect size**
Age	No	56.23	17.204	0.327	0.02	Insignificant
	Yes	60.90	14.928			
BMI^‡^	No	26.08	4.872	0.200	0.05	Insignificant
	Yes	23.80	3.768			
Mean pressure in the bag	No	274.80	30.800	0.046	0.70	Average
	Yes	255.75	34.372			
**Variable**	**Complication**	**Mean**	**Sum** **Rank**	**p-value** ^§^	**Pearson’s r**	**Effect size**
Usage time (days)	No	24.37	731	0.493	0.10	Insignificant
	Yes	27.20	544			

*SD = Standard deviation; ^†^T-test independent samples; ^‡^BMI = Body Mass Index; ^§^Mann-Whitney U test

**Table 3:** - Comparison between outcomes and categorical variables. São Carlos, SP, Brazil, 2022

**Risk factors**		**With complication**	**No complication**	**Total**	**p-value**	**RR and CI**
Sex	Female	10	12	22	0.485*	0.7 (0.400; 1.544)
	Male	10	18	28		
Use of sedation	Yes	13	20	33	0.903*	1.0 (0.515; 0.227)
	No	7	10	17		
Use of anticoagulant	Yes	7	11	18	0.904*	1.0 (0.511; 2.135)
	No	13	19	32		
Use of antibiotics	Yes	15	22	37	0.895*	0.9 (0.430; 2.092)
	No	5	8	13		
Eating habits	Oral route	9	12	21	0.726*	1.1 (0.573; 2.227)
	Nasoenteric tube	11	18	29		
Puncture site	Radial	17	26	43	1,000^†^	0.9 (0.363; 2.342)
	Dorsalis Pedis	3	4	7		
Type of bandage	Gauze and micropore	6	12	18	0.470*	1.3 (0.612; 2.813)
	Transparent waterproof	14	18	32		
Volume of the solution	≤500 ml	4	10	14	0.353^†^	0.6 (0.260; 1.589)
	1,000 ml	16	20	36		
Catheter gauge size	18G	7	14	21	0.413*	1.3 (0.650; 2.783)
	20G; 22G	13	16	29		

*Pearson’s Chi-square; ^†^Fisher’s Exact Test

When assessing the relationship between the outcome and age, time of device usage and level of pressure used in the pressurizing bag system, it is noted that the pressure in the bag was associated with the catheter being removed before the indicated time (p=0.046), with pressures below the appropriate level being related to losses and removals before the scheduled time (Table 2). Furthermore, the magnitude of this difference can be considered of medium intensity by the Hedges’ g value.

## Discussion

This study listed the early removal of the device due to obstruction (36.54%) and phlebitis (52.00%) as the main complications found. The literature describes local pain, paresthesia, hematoma, bleeding, ischemic complications, embolism, vascular thrombosis and occlusion, vascular injury, compartment syndrome, pseudoaneurysm formation, abscess, infection, sepsis, and local nerve injury as IBP complications^(1-4,9)^. Among these, the most common are vascular occlusion, bleeding at the insertion site, and hematoma formation. However, although more frequent, these complications can be identified and treated effectively, which reduces the possibility of major complications^(4)^.

Another complication described in the literature refers to the use of invasive blood pressure monitoring as a factor that increased the chances of myocardial injury compared to patients monitored with non-invasive blood pressure techniques^(17)^.

A literature review evaluated 19,617 punctures in the radial artery and described temporary occlusion of the artery (incidence of 19.70%) as the main complication found at this puncture site. Also in this review, the second most used artery for hemodynamic monitoring was the femoral artery and temporary occlusion at this site was reported in only 1.18% of cases. Lower rates of this complication in the femoral artery can be attributed to the larger diameter of this vessel. Other complications described by these authors such as ischemic damage, sepsis, and pseudoaneurysm formation showed similar results for the radial, femoral, and axillary arteries^(2)^.

It was also identified that most punctures were performed in the radial artery (86%). This data corroborates the findings of a literature review that mentioned the radial artery as the most used site for installing the invasive blood pressure monitoring device. According to the authors, this occurs due to its low complication rate and easy access to this site^(2)^.

In this study, the factor associated with early removal of the device was the level of pressure in the bag (p=0.046), with lower values being related to a greater number of device losses. It is recommended that pressure values be close to 300 mmHg to maintain adequate device permeability^(3,18)^. In the present study, the mean value found in cases of device removal before the recommended time was 262.22 mmHg.

It is known that maintaining adequate pressure levels in the IBP device is essential to ensure access permeability, and the pressure exerted aims to prevent blood reflux and catheter obstruction. In this sense, some references suggest the use of heparin as a method to prevent thrombus formation^(19)^. In the present study, heparin was not used in the saline solution of any device.

Another finding in the literature shows that pre- or post-procedure anticoagulation, as well as the use of vasodilators and local compression, reduce the risk of arterial occlusion after arterial puncture^(9)^. However, in this study, no association was found between the use of systemic anticoagulants (p=0.745) and complication outcomes, as observed in randomized and systematic review studies already published^(9,20-21)^.

A study conducted with patients facing sepsis aimed to evaluate possible differences in invasive and noninvasive blood pressure measurements among patients with septic shock, and it identified an association between the use of vasopressors and increased likelihood of a clinically significant blood pressure discrepancy when comparing invasive and noninvasive measurements. A higher SOFA score and higher serum lactate levels were also associated with a greater likelihood of blood pressure discrepancy, leading to clinical changes in the treatment^(22)^. The present study intended to evaluate the relationship between the use of vasoactive drugs and its complications; however, due to the limited number of participants who were not using these medications, this analysis was not performed.

Still in the present study, the most prevalent complication was phlebitis, which led to an early removal of the device in 52% of cases. A cohort study that evaluated and documented cases of phlebitis in venous accesses found an incidence of this complication in 43.2% of cases and indicated the length of hospital stay and the number of catheters inserted as related factors^(23)^. In turn, a multicenter cohort study evaluated 3,429 peripheral catheters in critically ill patients and found the following main risk factors for the development of phlebitis: institutional factors, factors inherent to the patient, the type of catheter, and drug-induced factors^(24)^. It is noteworthy that these studies evaluated phlebitis in venous access, while few studies evaluated this relationship in arterial catheters.

Regarding phlebitis in arterial accesses, a review study^(9)^ revealed that arterial catheterization presents low risk of infection, with an incidence of less than 1%. Furthermore, it described that infections in arterial accesses may be related to the development of pseudoaneurysm in the punctured vessel, as reported in another review, according to which infections in this type of access are rare^(25)^.

The incidence of infections in arterial accesses can be reduced by implementing certain methods such as aseptic insertion technique and adequate disinfection of the insertion site. However, prophylactic antibiotic therapy does not seem to reduce the risk of catheter-related infections^(2)^.

One of the factors related to a higher risk of infectious complications and sepsis is the permanence of the arterial catheter, especially if the puncture has been placed for more than 96 hours^(2)^. In this study, the average catheter dwell-time was 4.36 days (SD: 3.504), with a minimum of 1 day and a maximum of 16.

The catheter material is also described as a factor to be considered, since some materials seem to be more resistant to certain microorganisms^(2)^. In this study, however, this data could not be evaluated, since only one type of catheter was used to perform punctures.

The study had limitations since it was developed in a single hospital, which made it impossible to compare it with other protocols for the use of invasive blood pressure (IBP), such as other types of catheter, different kinds of bandages, fixation methods, puncture techniques or more diverse puncture sites. In addition, convenience sampling can introduce bias in the analysis.

There was interest in verifying whether the use of vasoactive drugs would impact the incidence of complications; however, given the low frequency of participants who did not use these medications, it was not possible to obtain a reliable result in this regard. This can also be described as a limitation of the study. It is suggested that this variable be evaluated again in future research studies, with larger samples.

The study’s contributions include the importance of adequately maintaining pressure in the monitoring device bag. This is a nursing care measure that is simple to assess and implement, capable of reducing complications. Furthermore, as phlebitis was the main complication found, the need for rigorous care to prevent infections associated with the puncture site is highlighted.

## Conclusion

The factor related to complications of the IBP monitoring system found in the present study refers to the levels of pressure in the bag, which was associated with the removal of the catheter before the indicated time. The main complications associated with catheter removal before the indicated time were phlebitis and obstruction.
