# Supplementary material for: Factors related to complications of the invasive blood pressure system among adult and elderly patients: a prospective study
Source: Rev Lat Am Enfermagem. 2025 Jan 31;33:e4443. doi: 10.1590/1518-8345.7097.4443 (PMC11789755; doi:10.1590/1518-8345.7097.4443)
Supplement: Translation

# Translation: Factores relacionados con complicaciones en el sistema de presión arterial invasiva en pacientes adultos y adultos mayores: estudio prospectivo

**DOI:** 10.1590/1518-8345.7097.4442

**Authors:** Willian Marques de Souza, Victor Hugo Baldan Cypriano, Rosângela Aparecida de Sousa, Rafael Luis Bressani Lino, Ana Luiza Mroczinski, Danielle Cristina Garbuio

**Affiliations:** 1 Centro Universitário Central Paulista, Departamento de Enfermagem, São Carlos, SP, Brasil.; 2 Universidade Federal de São Carlos, Departamento de Enfermagem, São Carlos, SP, Brasil.; 3 Universidade de São Paulo, Escola de Enfermagem de Ribeirão Preto, PAHO/WHO Collaborating Centre for Nursing Research Development, Ribeirão Preto, SP, Brasil.

**Keywords:** Enfermería, Monitorización Hemodinámica, Presión Arterial, Atención de Enfermería, Enfermería de Práctica Avanzada, Enfermería de Cuidados Críticos

## Abstract

**Objetivo:**

identificar los principales factores relacionados con las complicaciones en el sistema invasivo de presión arterial.

**Método:**

estudio prospectivo, realizado con personas mayores de 18 años ingresadas en cuidados intensivos, utilizando un dispositivo para medir la presión arterial invasiva. Los participantes fueron monitoreados mientras tenían colocado el catéter y se recolectaron datos sociodemográficos, clínicos y del dispositivo. El resultado analizado fue el retiro por no indicación de uso o por complicación. Para los análisis se utilizaron la prueba t de Student, la prueba U de Mann-Whitney, la prueba de chi-cuadrado y la prueba de Fisher.

**Resultados:**

se incluyeron y monitorearon 50 participantes y la mayoría de los dispositivos fueron colocados en la arteria radial (86%), con catéter calibre 20 *gauge* (50%), todos con catéter flexible. Cada paciente pasó, en promedio, 4,36 días (DE: 3,504) con el dispositivo. En cuanto a los resultados, el 60,0% de los dispositivos fueron retirados por no indicación de uso y el 40,0% por complicaciones. La flebitis fue la complicación más prevalente y la presión de la bolsa fue el factor asociado al retiro del catéter antes del momento de la indicación (p=0,046).

**Conclusión:**

las principales complicaciones de este dispositivo fueron la obstrucción y la flebitis, mientras que la presión de la bolsa fue el factor relacionado con el retiro del catéter antes de la indicación.

## Highlights

**(1)** La flebitis fue la complicación más frecuente.

**(2)** La presión de la bolsa se asoció con el retiro del catéter antes de la indicación.

**(3)** El tiempo de permanencia y el uso de sedación se relacionaron con la aparición de flebitis.

## Introducción

La canulación arterial es un procedimiento frecuente en entornos de cuidados intensivos y críticos. Es un método invasivo para medir con precisión la presión arterial y la presión arterial media y, por ende, proporciona mediciones que permiten reconocer de inmediato los cambios para realizar una intervención rápida. Además, es un acceso menos traumático que las recolecciones frecuentes de sangre arterial^(1-4)^.

Se recomienda realizar este procedimiento en pacientes críticos con inestabilidad hemodinámica, como en emergencias hipertensivas, estados de shock, uso de aminas vasoactivas, vasodilatadores, vasopresores e inotrópicos y pacientes en el postoperatorio inmediato de cirugía cardíaca y neurológica. También está indicado en situaciones en las que el paciente no puede someterse a un monitoreo no invasivo de la presión arterial, en quemaduras de gran superficie corporal o en fracturas múltiples de las extremidades^(4-5)^.

Para obtener una medición y un análisis adecuados de los valores de presión arterial invasiva (PAI), el catéter debe insertarse, posicionarse y mantenerse adecuadamente. Inicialmente se elige el sitio de punción y el calibre del catéter a introducir y, después de la punción, es fundamental que el transductor esté nivelado sobre el eje flebostático, por último se realiza una observación crítica de la onda de la presión arterial en el monitor multiparamétrico^(3)^.

Los sitios de punción recomendados para monitorear la PAI o recolectar sangre arterial son las arterias: radial, dorsal del pie y femoral. Debido al fácil acceso y al bajo riesgo de contaminación y pérdida, la arteria radial es el sitio preferido para la punción y la inserción del dispositivo^(1,6)^. Por otro lado, en el caso de pacientes en shock refractario, el monitoreo de la PAI a través de la arteria radial produce valores de presión menos precisos que la punción femoral^(7-8)^. El tipo de catéter y calibre dependerá de varios factores, como la arteria a puncionar y los riesgos que implica el procedimiento^(3)^.

Para la canulación de la arteria radial se recomienda el Test de Allen, cuyo objetivo es evaluar la circulación colateral de la arteria cubital, para evitar lesión isquémica^(1)^. Sin embargo, los estudios demuestran que tiene baja precisión diagnóstica y no hay que considerarlo un predictor de complicaciones isquémicas en la punción de la arteria radial^(3)^.

Las contraindicaciones absolutas para la inserción de un catéter arterial incluyen falta de pulso, síndrome de Raynaud, circulación inadecuada o interrumpida e infección en el sitio de inserción. Los casos de trastornos de la coagulación y situaciones de anticoagulación, quemaduras, injertos arteriales e intervenciones quirúrgicas en el lugar de inserción deben evaluarse individualmente^(1,4)^.

En cuanto a las complicaciones relacionadas con la punción arterial para monitoreo invasivo, dependerán del sitio de inserción^(2,9)^ y, en general, se observa dolor en el sitio, además de parestesia, hematoma, sangrado, complicaciones isquémicas, embolias, trombosis vascular, oclusión, lesión de vaso, formación de pseudoaneurisma, absceso y lesión nerviosa local^(1-3,9)^. Si bien las complicaciones graves relacionadas con este procedimiento no son frecuentes, hay que realizar un análisis riesgo-beneficio para cada paciente^(4)^. Además, estudios recientes indican que la punción arterial guiada por ultrasonido tiene una relación significativa con la reducción de fracasos del procedimiento^(10)^.

En Brasil, dentro del equipo de enfermería, la punción arterial, tanto para la gasometría arterial como para el monitoreo hemodinámico, es una práctica exclusiva de los enfermeros. Por lo tanto, es fundamental que este profesional esté capacitado y calificado para instalar la PAI, e incluso, que pueda utilizar ultrasonido a pie de cama para ayudar con la punción y el uso del botón anestésico para fijar el catéter^(11)^.

En este caso, el enfermero debe tener conocimiento sobre la inserción de la PAI, las principales complicaciones del procedimiento y posibles factores predisponentes, para poder brindar cuidados de enfermería calificados y crear planes de cuidados específicos^(12)^. Por consiguiente, se elaboró la pregunta orientadora de este estudio: ¿Cuáles son los principales factores relacionados con las complicaciones en el sistema de PAI en pacientes adultos? Para responder esa pregunta, se estableció como objetivo identificar los principales factores relacionados con las complicaciones del sistema de monitoreo de PAI.

## Método

### Diseño del estudio

Estudio cuantitativo, observacional, prospectivo, orientado por el *Strengthening the Reporting of Observational Studies in Epidemiology* (STROBE)^(13)^.

### Lugar y periodo de recolección

La recolección de datos se realizó en julio y agosto de 2022, en un hospital filantrópico del interior del estado de São Paulo. El hospital cuenta con tres unidades de cuidados intensivos para adultos, una específica de cardiología con 15 camas y dos de cuidados generales, con 10 camas cada una. El estudio se llevó a cabo en esas tres unidades.

### Criterios de población y selección

Se incluyeron pacientes mayores de 18 años (adultos y adultos mayores), hospitalizados en las mencionadas unidades de cuidados intensivos, con indicación de uso de un dispositivo para medir la presión arterial invasiva (pacientes en shock, en uso de drogas vasoactivas, inestabilidad hemodinámica, cirugía mayor, gasometría frecuente, inotrópicos, crisis hipertensivas, entre otros)^(3)^ instalado en las últimas 48 horas en las unidades mencionadas. El nivel de conciencia no fue un criterio de elegibilidad, ya que, debido al estado de salud de los participantes, muchos podrían estar sedados o tener comprometido el nivel de conciencia. Por ello se decidió invitar a participar a los familiares.

### Recolección de datos y variables del estudio

La recolección se realizó mediante visitas diarias a las unidades en busca de participantes elegibles y para actualizar los datos de los que estaban siendo monitoreados. Cuando el paciente cumplía con los criterios de inclusión, se contactaba al familiar para presentarle la investigación, los objetivos y riesgos y, si aceptaba, se le solicitaba que firmara el Formulario de Consentimiento Libre e Informado (FCLI) para registrar el consentimiento.

Luego de obtener el consentimiento, se recolectaron de la historia clínica datos sociodemográficos sobre edad, sexo, raza, tabaquismo, consumo de alcohol, comorbilidades, tipo de dieta, índice de masa corporal (IMC), dispositivos y medicamentos que usaba. Se recolectaron datos relacionados con la PAI: sitios de punción, calibre y tipo de catéter, volumen de solución salina y tipo de apósito y presión en la bolsa. En los días siguientes se evaluó el tipo de apósito, la presión de la bolsa presurizadora, la permeabilidad del catéter y los signos de flebitis (utilizando la escala de flebitis visual de *Infusion Nursing Society* ^(14)^). Cuando se retiró el catéter, por indicación o complicación, se dio por terminada la participación en la investigación.

Las variables predictoras evaluadas fueron: edad, sexo, raza, tabaquismo, consumo de alcohol, comorbilidades, tipo de alimentación, IMC, dispositivos y medicamentos que utilizaba, tipo de apósito, sitios de punción, calibre y tipo de catéter, volumen de solución salina y tipo de apósito y presión en la bolsa. Los resultados evaluados fueron el retiro de este dispositivo antes de lo previsto por infección u obstrucción, muerte o por falta de indicación de uso.

Los participantes fueron monitoreados diariamente hasta que se les retiró el dispositivo por indicación, en caso de mejoría clínica, complicación o muerte. Por lo tanto, se consideró retiro programado el que estaba dentro de lo previsto y retiro por complicación cuando se realizó por infección, obstrucción o retiro accidental. Para realizar los análisis, el retiro por muerte se consideró por indicación.

### Procesamiento y análisis de los datos

Los datos recolectados se ingresaron en hojas de cálculo de *Microsoft Excel* ^®^ con doble entrada. Las variables se analizaron inicialmente mediante estadística descriptiva de cada variable y las distribuciones se verificaron mediante la prueba de Shapiro-Wilk; posteriormente se determinó la incidencia de las complicaciones. Para calcular la incidencia se realizó una división entre el número total de retiros por complicación y el número total de punciones. Se compararon las variables continuas que seguían una distribución normal de los grupos con y sin complicaciones mediante la prueba t de Student. El tamaño del efecto de la diferencia entre las medias se evaluó utilizando la g de Hedges, y se consideraron insignificantes los valores de referencia para la magnitud del efecto^(15)^ inferiores a 0,19 y medios los valores entre 0,50 y 0,79. La variable continua que no siguió una distribución normal se evaluó entre los grupos mediante la prueba U de Mann-Whitney y, en este caso, el tamaño del efecto se calculó mediante la r^(16)^ de Pearson. Para las variables categóricas, se realizó la correlación con el resultado mediante la prueba Chi-cuadrado de Pearson o la prueba Exacta de Fisher (si los valores en las columnas eran inferiores a 5). También se calcularon el riesgo relativo y los respectivos intervalos de confianza para estos análisis. Se utilizó el *software IBM SPSS Statistics 22* ^®^ considerando un nivel de significación (α) del 5%.

### Aspectos éticos

El proyecto de investigación fue sometido a evaluación del Comité de Ética en Investigación con Humanos y aprobado bajo dictamen 5.484.178 de 2022. Los acompañantes y participantes fueron abordados solamente de forma ética, para explicarles los propósitos de la investigación y confirmar sus respectivos consentimientos a través de la firma del Formulario de Consentimiento Libre e Informado (FCLI). Se siguieron todos los preceptos éticos determinados por la Resolución 466/12 del Consejo Nacional de Salud del Ministerio de Salud brasileño.

## Resultados

Durante el período de recolección de datos, se seleccionaron 88 participantes elegibles para la investigación. El flujo de selección e inclusión se presenta en la Figura 1.

**Figura 1:** - Diagrama de flujo de selección, inclusión y seguimiento de los participantes en la investigación. São Carlos, SP, Brasil, 2022

La edad promedio de los participantes era de 58,1 años (DE: 16.337), la mayoría eran hombres (56%), de raza blanca (60%), solteros (48%). La Tabla 1 presenta la caracterización sociodemográfica y clínica de los participantes.

**Tabla 1:** - Caracterización sociodemográfica, clínica e información sobre el sistema de presión arterial invasiva de los participantes (n = 50). São Carlos, SP, Brasil, 2022

	**N**	**%**
**Sexo** Masculino Femenino	28 22	56 44
**Raza/Color** Blanca Morena Negra	30 17 03	60 34 03
**Tabaquismo** Fumador No fumador Exfumador No identificado	08 21 14 07	16 42 28 14
**Consumo de alcohol** Consume No consume Consumía No identificado	16 19 04 11	32 38 08 22
Comorbilidades* Diabetes Mellitus Hipertensión Arterial Sistémica Enfermedad Renal Crónica Cardiopatías Otras	16 36 00 02 28	32 72 00 04 56
**Alimentación** Vía oral Sonda nasogástrica	21 29	42 58
**Droga vasoactiva** Sí No	48 02	96 04
**Sedación** Sí No	33 17	66 34
**Antibiótico** Sí No	37 13	74 26
**Anticoagulante** Sí No	18 32	36 64
**Catéter venoso central** Sí No	37 13	74 26
**Sitio de punción** Radial Del pie	43 07	86 14
**Volumen de solución presurizada** 250 ml 500 ml 1000 ml	1 13 36	02 26 72
**Apósito aplicado en el momento de la punción** Transparente e Impermeable Gasa y Microporo	32 18	64 36
**Motivos de retiro** No indicación - programado Complicación Muerte	24 20 06	48 40 12

*Cada paciente podría presentar más de una comorbilidad y la suma ser mayor a 50

Considerando las características de los dispositivos invasivos para medir la presión arterial, la mayoría de ellos se instalaron en la arteria radial (86%), todos mediante un catéter flexible sin aguja. Durante el período establecido para la recolección no se instaló ningún dispositivo en la arteria femoral. Cabe destacar que la prueba de Allen se realizó en el 86% de las punciones radiales. El catéter más utilizado fue el de calibre 20 *gauge* (G) (50%), seguido del 18G (42%) y, por último, el 22G (8%).

Cada paciente pasó, en promedio, 4,36 días (DE: 3,504) con el dispositivo, con un mínimo de 1 día y un máximo de 16. La incidencia de complicaciones alcanzó el 40% y, entre ellas, el 52% de las punciones mostraron signos de flebitis, el 36,54% signos de obstrucción y el 11,46% otras complicaciones. En todos los casos de complicación se retiró el catéter y no se continuó con el estudio tras la nueva punción.

Las variables edad (p=0,156), IMC (p=0,725) y presión media de la bolsa (p=0,153) presentaron distribución normal que fue evaluada mediante la prueba de Shapiro-Wilk. La variable tiempo de permanencia del catéter en días fue evaluada mediante la misma prueba y no presentó distribución normal (p<0,000).

En este estudio no se encontró relación entre el resultado (retiro por complicación o indicación) para las variables sexo, edad, tiempo de permanencia, uso de anticoagulantes, antibióticos o sedación, tipo de alimentación, calibre del catéter y otros factores, como se puede ver en las Tablas 2 y 3.

**Tabla 2:** - Comparación de los resultados con valores medios de edad, tiempo de uso del dispositivo y presión en la bolsa presurizadora (n = 50). São Carlos, SP, Brasil, 2022

**Variable**	**Complicación**	**Media**	DE*	**p valor** ^†^	**g de Hedges**	**Tamaño del efecto**
Edad	No	56,23	17,204	0,327	0,02	Insignificante
	Sí	60,90	14,928			
IMC^‡^	No	26,08	4,872	0,200	0,05	Insignificante
	Sí	23,80	3,768			
Presión media bolsa	No	274,80	30,800	0,046	0,70	Medio
	Sí	255,75	34,372			
**Variable**	**Complicación**	**Postos Médios**	**Suma Clasificación**	**p valor** ^§^	**r de Pearson**	**Tamaño del efecto**
Tiempo de uso (días)	No	24,37	731	0,493	0,10	Insignificante
	Sí	27,20	544			

*DE = Desviación estándar; ^†^Prueba t de muestras independientes; ^‡^IMC = Índice de Masa Corporal; ^§^Prueba U de Mann-Whitney

**Tabla 3:** - Comparación de los resultados con las variables categóricas. São Carlos, SP, Brasil, 2022

**Factor de riesgo**		**Con Complicación**	**Sin Complicación**	**Total**	**p valor**	**RR e IC**
Sexo	Femenino	10	12	22	0,485*	0,7 (0,400; 1,544)
	Masculino	10	18	28		
Uso de sedación	Sí	13	20	33	0,903*	1,0 (0,515; 0,227)
	No	7	10	17		
Uso de anticoagulante	Sí	7	11	18	0,904*	1,0 (0,511; 2,135)
	No	13	19	32		
Uso de antibiótico	Sí	15	22	37	0,895*	0,9 (0,430; 2,092)
	No	5	8	13		
Alimentación	Vía oral	9	12	21	0,726*	1,1 (0,573; 2,227)
	SNE	11	18	29		
Sitio de punción	Radial	17	26	43	1,000^†^	0,9 (0,363; 2,342)
	Del pie	3	4	7		
Tipo de apósito	Gasa y cinta microporosa	6	12	18	0,470*	1,3 (0,612; 2,813)
	Transparente impermeable	14	18	32		
Volumen de la solución	≤500ml	4	10	14	0,353^†^	0,6 (0,260; 1,589)
	1.000ml	16	20	36		
Calibre del catéter	18G	7	14	21	0,413*	1,3 (0,650; 2,783)
	20G; 22G	13	16	29		

*Chi-cuadrado de Pearson; ^†^Prueba Exacta de Fisher

Al evaluar la relación entre el resultado y la edad, tiempo de uso del dispositivo y presión utilizada en el sistema de bolsa presurizadora, se observa que la presión de la bolsa se asoció con el retiro del catéter antes de lo indicado (p=0,046), mientras que las presiones por debajo de lo adecuado se relacionan con pérdidas y retiros antes de lo previsto (Tabla 2). Además, la magnitud de esta diferencia se puede considerar de intensidad media por el valor de g de Hedges.

## Discusión

Este estudio determinó que las principales complicaciones por las que se retiró tempranamente el dispositivo eran obstrucción (36,54%) y flebitis (52,00%). La literatura describe complicaciones de la PAI como dolor local, parestesia, hematoma, sangrado, complicaciones isquémicas, embolia, trombosis vascular y oclusión, lesión vascular, síndrome compartimental, formación de pseudoaneurisma, absceso, infección, sepsis y lesión nerviosa local^(1-4,9)^. Entre ellas, las más comunes son oclusión del vaso, sangrado en el sitio de inserción y formación de hematomas. Sin embargo, a pesar de ser más frecuentes, estas complicaciones pueden identificarse y tratarse eficazmente, lo que reduce la posibilidad de complicaciones mayores^(4)^.

Otra complicación descrita en la literatura es que el uso de monitoreo invasivo de la presión arterial tiene más posibilidades de provocar lesión miocárdica que el monitoreo mediante presión arterial no invasiva^(17)^.

Una revisión de la literatura evaluó 19.617 punciones en la arteria radial y concluyó que la oclusión temporal de la arteria (incidencia del 19,70%) era la principal complicación de este sitio de punción. En esta revisión, la segunda arteria más utilizada para el monitoreo hemodinámico fue la arteria femoral y, en ese sitio, la oclusión temporal se informó en sólo el 1,18% de los casos. Las tasas más bajas de dicha complicación en la arteria femoral se pueden atribuir a que este vaso tiene mayor diámetro. Otras complicaciones descritas por estos autores, como daño isquémico, sepsis y formación de pseudoaneurismas, fueron similares en las arterias radial, femoral y axilar^(2)^.

También se identificó que la mayoría de las punciones se realizó en la arteria radial (86%). Este dato coincide con lo encontrado en una revisión de la literatura, que señaló que la arteria radial era el sitio más utilizado para instalar el dispositivo de monitoreo de la presión arterial invasiva. Según los autores, esto se debe a la baja tasa de complicaciones y al fácil acceso a ese sitio^(2)^.

En este estudio, el factor que demostró relación con el retiro temprano del dispositivo fue la presión de la bolsa (p=0,046), dado que los valores más bajos se relacionaban con un mayor número de pérdidas del dispositivo. Se recomienda que los valores de presión sean cercanos a 300 mmHg para mantener una adecuada permeabilidad del dispositivo^(3,18)^. En el presente estudio, el valor medio observado en los casos con retiro del dispositivo antes de lo recomendado fue de 262,22 mmHg.

Se sabe que mantener niveles de presión adecuados en el dispositivo de PAI es un cuidado fundamental para la permeabilidad del acceso, y el objetivo de la presión ejercida es prevenir el reflujo sanguíneo y la obstrucción del catéter. En este sentido, algunas referencias indican el uso de heparina como método para prevenir la formación de trombos^(19)^. En el presente estudio, no se utilizó heparina en la solución salina de ningún dispositivo.

Otro dato encontrado en la literatura indica que el uso de anticoagulantes antes o después del procedimiento, así como el uso de vasodilatadores y compresión local, reducen el riesgo de oclusión arterial después de la punción arterial^(9)^. Sin embargo, en este estudio no se observó que hubiera relación entre el uso de anticoagulantes sistémicos (p=0,745) y los resultados de la complicación, como se observó en estudios aleatorizados y de revisión sistemática publicados previamente^(9,20-21)^.

Un estudio realizado con pacientes con sepsis tuvo como objetivo evaluar posibles diferencias en las mediciones de presión arterial invasivas y no invasivas en pacientes con shock séptico e identificó una asociación entre el uso de vasopresores y una mayor probabilidad de discrepancia de presión arterial clínicamente significativa entre las medidas invasivas y no invasivas. Una puntuación más alta el puntaje SOFA y niveles más altos de lactato sérico también se asociaron con una mayor probabilidad de discrepancia en la presión arterial, lo que llevó a cambios clínicos en el tratamiento^(22)^. En el presente estudio se procuró evaluar la relación entre el uso de fármacos vasoactivos y las complicaciones; sin embargo, debido al número limitado de participantes que no utilizaron estos medicamentos, dicho análisis no se realizó.

Por otro lado, en el presente estudio, la complicación más prevalente fue la flebitis, responsable del retiro temprano del dispositivo en el 52% de los casos. Una cohorte que evaluó y documentó casos de flebitis en el acceso venoso indicó que la incidencia de esta complicación era del 43,2% de los casos y señaló que los factores relacionados eran tiempo de hospitalización y número de catéteres insertados^(23)^. Un estudio de cohorte multicéntrico evaluó 3.429 catéteres periféricos en pacientes críticos, y comprobó que los principales factores de riesgo para el desarrollo de flebitis eran: institucionales, inherentes al paciente, tipo de catéter y factores inducidos por fármacos^(24)^. Cabe destacar que estos estudios evaluaron la flebitis en el acceso venoso y que son pocos los estudios que evaluaron esa relación en los catéteres arteriales.

Con respecto a la flebitis en el acceso arterial, un estudio de revisión^(9)^ señaló que el cateterismo arterial presenta un bajo riesgo de infección, con una incidencia inferior al 1%. Además, describió que las infecciones en los accesos arteriales pueden estar relacionadas con el desarrollo de pseudoaneurisma en el vaso puncionado, como lo indica otra revisión, según la cual las infecciones en este tipo de accesos son raras^(25)^.

La reducción de la incidencia de infecciones en el acceso arterial se puede dar al implementar ciertos factores, como una técnica de inserción aséptica y una desinfección adecuada del sitio de inserción. Sin embargo, la terapia antibiótica profiláctica no parece reducir el riesgo de infecciones relacionadas con el catéter^(2)^.

Uno de los factores relacionados con un mayor riesgo de complicaciones infecciosas y sepsis es la permanencia del catéter arterial, especialmente si la punción permanece colocada por más de 96 horas^(2)^. En este estudio, el tiempo medio de permanencia del catéter fue de 4,36 días (DE: 3,504), con un mínimo de 1 día y un máximo de 16.

El material del catéter también se describe como un factor a considerar, ya que algunos materiales parecen ser más resistentes a ciertos microorganismos^(2)^. En el presente estudio, sin embargo, estos datos no pudieron ser evaluados, porque se utilizó un solo tipo de catéter en las punciones.

Las limitaciones del estudio fueron el hecho de que se desarrolló en un solo hospital, lo que impidió que se realizara una comparación con otros protocolos de uso de presión arterial invasiva (PAI), como otros catéteres, diferentes tipos de apósitos, formas de fijación, técnicas para punción o mayor diversidad de sitios de punción. Además, la muestra por conveniencia permite que haya sesgos en el análisis.

Se procuraba verificar si el uso de fármacos vasoactivos tendría efecto en la incidencia de complicaciones, sin embargo, debido a la baja frecuencia de participantes que no utilizaron estos medicamentos, no fue posible obtener un resultado confiable al respecto. Lo que también se puede considerar una limitación del estudio. Se sugiere evaluar nuevamente esa variable en futuras investigaciones, con muestras más grandes.

El estudio contribuye a demostrar la importancia de mantener adecuadamente la presión en la bolsa del dispositivo de monitoreo. Se trata de cuidados de enfermería fáciles de evaluar y realizar, que pueden reducir las complicaciones. Además, como la flebitis fue la principal complicación observada, se destaca que es necesario realizar cuidados estrictos para prevenir infecciones asociadas al sitio de punción.

## Conclusión

El factor relacionado con las complicaciones del sistema de monitoreo de la PAI que se encontró en el presente estudio fue la presión de la bolsa, que se asoció con el retiro del catéter antes del momento indicado. Las principales complicaciones asociadas al retiro del catéter antes de lo indicado fueron flebitis y obstrucción.

# Translation: Fatores relacionados às complicações no sistema de pressão arterial invasiva em pacientes adultos e idosos: estudo prospectivo

**DOI:** 10.1590/1518-8345.7097.4444

**Authors:** Willian Marques de Souza, Victor Hugo Baldan Cypriano, Rosângela Aparecida de Sousa, Rafael Luis Bressani Lino, Ana Luiza Mroczinski, Danielle Cristina Garbuio

**Affiliations:** 1 Centro Universitário Central Paulista, Departamento de Enfermagem, São Carlos, SP, Brasil.; 2 Universidade Federal de São Carlos, Departamento de Enfermagem, São Carlos, SP, Brasil.; 3 Universidade de São Paulo, Escola de Enfermagem de Ribeirão Preto, PAHO/WHO Collaborating Centre for Nursing Research Development, Ribeirão Preto, SP, Brasil.

**Keywords:** Enfermagem, Monitorização Hemodinâmica, Pressão Arterial, Cuidados de Enfermagem, Prática Avançada de Enfermagem, Enfermagem de Cuidados Críticos

## Abstract

**Objetivo:**

identificar os principais fatores relacionados com as complicações no sistema de pressão arterial invasiva.

**Método:**

estudo prospectivo, conduzido com maiores de 18 anos internados em terapia intensiva, utilizando dispositivo para mensuração da pressão arterial invasiva. Os participantes foram acompanhados durante a permanência do cateter e fez-se coleta de dados sociodemográficos, clínicos e do dispositivo. O desfecho analisado foi a retirada por não indicação de uso ou por complicação. Para as análises, foram utilizados teste t de Student, U de Mann-Whitney, qui-quadrado e teste de Fisher.

**Resultados:**

foram incluídos e acompanhados 50 participantes e a maioria dos dispositivos foi instalada na artéria radial (86%), com cateter calibre 20 *gauge* (50%), todos com cateter flexível. Cada paciente permaneceu, em média, 4,36 dias (dp: 3,504) com o dispositivo. Quanto aos desfechos, 60,0% dos dispositivos foram retirados por não indicação de uso e 40,0% devido às complicações. A flebite foi a complicação mais prevalente e a pressão da bolsa o fator associado à retirada do cateter antes do momento da indicação (p=0,046).

**Conclusão:**

as principais complicações deste dispositivo foram obstrução e flebite enquanto a pressão da bolsa constituiu o fator relacionado à retirada do cateter antes da indicação.

## Highlights

**(1)** A flebite foi a complicação mais prevalente.

**(2)** A pressão da bolsa teve associação com a retirada do cateter antes da indicação.

**(3)** Tempo de permanência e uso de sedação estavam relacionados ao surgimento de flebite.

## Introdução

A canulação arterial é um procedimento frequente em ambientes de cuidados agudos e críticos. Trata-se de um método invasivo para medir com precisão a pressão arterial e a pressão arterial média e, assim, fornece medidas que permitem o reconhecimento imediato das alterações, para uma rápida intervenção. Além disso, oferece acesso menos traumático a coletas frequentes de sangue arterial^(1-4)^.

Recomenda-se a realização deste procedimento para pacientes graves em instabilidade hemodinâmica, como em emergências hipertensivas, estados de choque, uso de aminas vasoativas, vasodilatadores, vasopressores e inotrópicos e pacientes em pós-operatório imediato de cirurgia cardíaca e neurológica. É também indicado para situações nas quais o paciente não pode ser submetido ao monitoramento não invasivo da pressão arterial, em queimaduras de grande área corporal ou fraturas múltiplas de extremidades^(4-5)^.

Para uma adequada mensuração e análise dos valores de pressão arterial invasiva (PAI), o cateter deve ser inserido, posicionado e mantido adequadamente. Inicialmente, realiza-se a escolha do local de punção e do calibre do cateter a ser introduzido e, após punção, é fundamental que o transdutor seja nivelado no eixo flebostático, finalizando com observação crítica da onda de pressão arterial no monitor multiparâmetros^(3)^.

Os sítios de punções recomendados para monitorização da PAI ou coleta de sangue arterial são as artérias radial, pediosa e femoral. Devido ao fácil acesso e baixo risco de contaminação e perda, a artéria radial é o local preferencial de punção e inserção do dispositivo^(1,6)^. Em contrapartida, no caso de pacientes em choque refratário, a monitorização da PAI por meio da artéria radial emite valores de pressão menos precisos, quando comparada à punção femoral^(7-8)^. Já o tipo de cateter e calibre dependerá de diversos fatores, como a artéria a ser puncionada e os riscos que envolvem o procedimento^(3)^.

Para a canulação da artéria radial, indica-se a realização do Teste de Allen, cujo objetivo é avaliar a circulação colateral da artéria ulnar, a fim de evitar lesão isquêmica^(1)^. Entretanto, estudos demonstram que ele apresenta baixa acurácia diagnóstica e não deve ser considerado um preditor de complicações isquêmicas na punção da artéria radial^(3)^.

As contraindicações absolutas para a inserção de um cateter arterial incluem ausência de pulso, síndrome de Raynaud, circulação inadequada ou interrompida e infecção no local de inserção. Os casos de distúrbios de coagulação e situações de anticoagulação, queimaduras, enxertos arteriais e intervenções cirúrgicas no local da inserção devem ser avaliados de forma individual^(1,4)^.

Em relação às complicações relacionadas com a punção arterial para fins de monitorização invasiva, dependerão do sítio de inserção^(2,9)^ e, em geral, observa-se dor no local, bem como parestesia, hematoma, sangramento, complicações isquêmicas, embolia, trombose vascular, oclusão, lesão do vaso, formação de pseudoaneurisma, abscesso e lesão nervosa local^(1-3,9)^. As complicações graves relacionadas a esse procedimento não são frequentes, mas uma análise de risco-benefício deve ser realizada para cada paciente^(4)^. Ademais, estudos recentes evidenciam que a punção arterial guiada por ultrassom está significativamente relacionada à redução de falhas no procedimento^(10)^.

No Brasil, dentro da equipe de enfermagem, a punção arterial, tanto para coleta de gasometria arterial quanto para fins de monitorização hemodinâmica, é prática privativa do enfermeiro. Dessa forma, torna-se essencial que este profissional seja capacitado e habilitado para a instalação da PAI, inclusive com a possibilidade de utilizar a ultrassonografia à beira-leito para o auxílio da punção e da realização do botão anestésico para fixação do cateter^(11)^.

Nesse contexto, o enfermeiro deve ter conhecimento sobre a inserção da PAI, principais complicações deste procedimento e possíveis fatores predisponentes, a fim de oferecer uma assistência de enfermagem qualificada e desenvolver planos de cuidados direcionados^(12)^. Diante do exposto, elaborou-se a seguinte pergunta norteadora para este estudo: Quais os principais fatores relacionados às complicações no sistema de PAI no paciente adulto? Para respondê-la, definiu-se o objetivo de identificar os principais fatores relacionados às complicações do sistema de monitorização de PAI.

## Método

### Delineamento do estudo

Estudo quantitativo, observacional, prospectivo, orientado pelo *Strengthening the Reporting of Observational Studies in Epidemiology* (STROBE)^(13)^.

### Local e período da coleta

A coleta de dados foi realizada nos meses de julho e agosto de 2022, em um hospital filantrópico no interior do estado de São Paulo. O hospital possui três unidades de terapia intensiva adulto, sendo uma específica para cardiologia com 15 leitos e duas para atendimentos gerais, cada qual com 10 leitos. O estudo foi desenvolvido nessas três unidades.

### População e critérios de seleção

Foram incluídos pacientes maiores de 18 anos (adultos e idosos), internados nas referidas unidades de terapia intensiva, com indicação de uso de dispositivo para mensuração da pressão arterial invasiva (pacientes em choque, em uso de droga vasoativa, instabilidade hemodinâmica, cirurgia de grande porte, coleta frequente de gasometria, drogas inotrópicas, crises hipertensivas, entre outras)^(3)^ instalado nas últimas 48 horas nas referidas unidades. O nível de consciência não foi um critério de elegibilidade, uma vez que, em decorrência do estado de saúde dos participantes, muitos poderiam estar sedados ou com comprometimento do nível de consciência. Desse modo, optou-se por direcionar o convite para a participação aos familiares.

### Coleta de dados e variáveis do estudo

A coleta foi realizada mediante visitas diárias nas unidades em busca de participantes elegíveis e para atualização dos dados daqueles em acompanhamento. Quando o paciente atendia aos critérios de inclusão, contatava-se o familiar, a fim de apresentar a pesquisa, objetivos, riscos e, em caso de aceite, solicitava-se assinatura no Termo de Consentimento Livre e Esclarecido (TCLE), para registro da anuência.

Após o aceite, eram coletados do prontuário dados sociodemográficos de idade, sexo, raça, tabagismo, etilismo, comorbidades, tipo de alimentação, Índice de Massa Corporal (IMC), dispositivos e medicamentos em uso. Constituíram dados relacionados a PAI coletados: locais de punção, calibre e tipo do cateter, volume de solução fisiológica e tipo de curativo e pressão na bolsa. Nos dias subsequentes, eram avaliados tipo de curativo, pressão da bolsa pressurizadora, permeabilidade do cateter e sinais de flebite (utilizando a escala visual de flebite da *Infusion Nursing Society* ^(14)^). Quando o cateter era removido, por indicação ou por complicação a participação na pesquisa era encerrada.

As variáveis preditoras avaliadas foram: idade, sexo, raça, tabagismo, etilismo, comorbidades, tipo de alimentação, IMC, dispositivos e medicamentos em uso, tipo de curativo, locais de punção, calibre e tipo do cateter, volume de solução fisiológica e tipo de curativo e pressão na bolsa. Já os desfechos avaliados foram as retiradas deste dispositivo antes do programado por infecção ou obstrução, por óbito ou, ainda, pela não indicação de uso.

Os participantes foram acompanhados diariamente até a retirada do dispositivo por indicação, em caso de melhora clínica, por complicação ou óbito. Dessa forma, considerou-se como retirada prevista aquela dentro do programado e retirada por complicação quando feita por motivo de infecção, obstrução ou retirada acidental. Para as análises, considerou-se a retirada por óbito como retirada por indicação.

### Tratamento e análise dos dados

Os dados coletados foram digitados em planilhas *Microsoft Excel* ^®^ com dupla digitação. As variáveis foram analisadas inicialmente por meio de estatística descritiva de cada variável e verificadas suas distribuições pelo teste de Shapiro-Wilk; após, determinou-se a incidência das complicações. Para o cálculo da incidência, o número total de retiradas por complicação foi dividido pelo quantitativo total de punções. As variáveis contínuas que seguiram distribuição normal foram comparadas para os grupos com e sem complicação por meio do teste t de Student. Avaliou-se o tamanho do efeito da diferença entre as médias por meio do g de Hedges, considerando os valores de referência para a magnitude de efeito^(15)^ como insignificantes para os menores de 0,19 e médios para os valores entre 0,50 e 0,79. A variável contínua que não seguiu distribuição normal foi avaliada entre os grupos utilizando o teste U de Mann-Whitney e, nesse caso, calculou-se o tamanho do efeito por meio do r de Pearson^(16)^. Para variáveis categóricas, fez-se a correlação com o desfecho pelo teste de Qui-quadrado de Pearson ou teste Exato de Fisher (caso os valores nas colunas fossem menores que 5). Foram também calculados o risco relativo e os respectivos intervalos de confiança para essas análises. O *software* *IBM* *SPSS Statistics* 22^®^ foi utilizado considerando nível de significância (α) de 5%.

### Aspectos éticos

O projeto de pesquisa foi submetido à apreciação do Comitê de Ética em Pesquisa em Seres Humanos e aprovado sob parecer 5.484.178 de 2022. Os acompanhantes e participantes apenas foram abordados eticamente, para explicitação dos propósitos da pesquisa e afirmação das respectivas anuências através de assinatura no Termo de Consentimento Livre e Esclarecido (TCLE). Todos os preceitos éticos determinados pela Resolução 466/12 do Conselho Nacional de Saúde do Ministério de Saúde foram seguidos.

## Resultados

Durante o período da coleta de dados foram elencados 88 participantes elegíveis para pesquisa. O fluxo de rastreio e inclusão está apresentado na Figura 1.

**Figura 1:** - Fluxograma de rastreio, inserção e seguimento dos participantes na pesquisa. São Carlos, SP, Brasil, 2022

A média de idade dos participantes foi de 58,1 anos (dp: 16,337), a maioria homens (56%), da raça branca (60%), solteiros (48%). A Tabela 1 apresenta a caracterização sociodemográfica e clínica dos participantes.

**Tabela 1:** - Caracterização sociodemográfica, clínica e informações sobre o sistema de pressão arterial invasiva dos participantes (n = 50). São Carlos, SP, Brasil, 2022

	**N**	**%**
**Sexo** Masculino Feminino	28 22	56 44
**Raça/Cor** Branca Parda Negra	30 17 03	60 34 03
**Tabagismo** Tabagista Não tabagista Ex-tabagista Não identificado	08 21 14 07	16 42 28 14
**Etilismo** Etilista Não etilista Ex-etilista Não identificado	16 19 04 11	32 38 08 22
Comorbidades* Diabetes Mellitus Hipertensão Arterial Sistêmica Doença Renal Crônica Cardiopatias Outros	16 36 00 02 28	32 72 00 04 56
**Alimentação** Via oral Sonda nasoentérica	21 29	42 58
**Droga vasoativa** Sim Não	48 02	96 04
**Sedação** Sim Não	33 17	66 34
**Antibiótico** Sim Não	37 13	74 26
**Anticoagulante** Sim Não	18 32	36 64
**Cateter venoso central** Sim Não	37 13	74 26
**Local da punção** Radial Pediosa	43 07	86 14
**Volume da solução pressurizadora** 250 ml 500 ml 1000 ml	1 13 36	02 26 72
**Curativo realizado no momento da punção** Transparente e Impermeável Gaze e Micropore	32 18	64 36
**Motivos de retirada** Não indicação - programada Complicação Óbito	24 20 06	48 40 12

*Cada paciente poderia apresentar mais de uma comorbidade, sendo a somatória superior a 50

Considerando as características dos dispositivos de mensuração de pressão arterial invasiva, a maior parte deles foi instalada na artéria radial (86%), todos utilizando o cateter não agulhado flexível. No período estabelecido para coleta, não houve dispositivo instalado na artéria femoral. Destaca-se que em 86% das punções radiais foi realizado o teste de Allen. O cateter mais utilizado foi o de calibre 20 *gauge* (G) (50%), seguido pelo de calibre 18G (42%) e, por último, o de 22G (8%).

Cada paciente permaneceu, em média, 4,36 dias (dp: 3,504) com o dispositivo, sendo o mínimo de 1 dia e o máximo de 16. A incidência de complicações chegou a 40% e, dentre elas, 52% das punções apresentaram sinais de flebite, 36,54% sinais de obstrução e 11,46% outras complicações. Em todos os casos de complicação, o cateter foi removido e o estudo não seguiu após a nova punção.

As variáveis idade (p=0,156), IMC (p=0,725) e pressão média da bolsa (p=0,153) apresentaram distribuição normal avaliada pelo teste de Shapiro-Wilk. Já a variável tempo de permanência do cateter, em dias, foi avaliada por meio do mesmo teste e não apresentou distribuição normal (p<0,000).

Neste estudo, não foi encontrada relação entre o desfecho (retirada por complicação ou por indicação) para as variáveis sexo, idade, tempo de permanência, uso de anticoagulantes, antibiótico ou sedação, tipo de alimentação, calibre do cateter e outros fatores, conforme exposto nas Tabelas 2 e 3.

**Tabela 2:** - Comparação dos desfechos com os valores médios de idade, tempo de uso do dispositivo e pressão na bolsa pressurizadora (n = 50). São Carlos, SP, Brasil, 2022

**Variável**	**Complicação**	**Média**	DP*	**p valor** ^†^	**g de Hedges**	**Tamanho do efeito**
Idade	Não	56,23	17,204	0,327	0,02	Insignificante
	Sim	60,90	14,928			
IMC^‡^	Não	26,08	4,872	0,200	0,05	Insignificante
	Sim	23,80	3,768			
Pressão média bolsa	Não	274,80	30,800	0,046	0,70	Médio
	Sim	255,75	34,372			
**Variável**	**Complicação**	**Postos de Média**	**Soma Classificação**	**p valor** ^§^	**r de Pearson**	**Tamanho do efeito**
Tempo de uso (dias)	Não	24,37	731	0,493	0,10	Insignificante
	Sim	27,20	544			

*DP = Desvio-Padrão; ^†^Teste t amostras independentes; ^‡^IMC = Índice de Massa Corporal; ^§^Teste U de Mann-Whitney

**Tabela 3:** - Comparação dos desfechos com as variáveis categóricas. São Carlos, SP, Brasil, 2022

**Fator de risco**		**Com complicação**	**Sem complicação**	**Total**	**p valor**	**RR e IC**
Sexo	Feminino	10	12	22	0,485*	0,7 (0,400; 1,544)
	Masculino	10	18	28		
Uso de sedação	Sim	13	20	33	0,903*	1,0 (0,515; 0,227)
	Não	7	10	17		
Uso de anticoagulante	Sim	7	11	18	0,904*	1,0 (0,511; 2,135)
	Não	13	19	32		
Uso de antibiótico	Sim	15	22	37	0,895*	0,9 (0,430; 2,092)
	Não	5	8	13		
Alimentação	Via oral	9	12	21	0,726*	1,1 (0,573; 2,227)
	SNE	11	18	29		
Local de punção	Radial	17	26	43	1,000^†^	0,9 (0,363; 2,342)
	Pediosa	3	4	7		
Tipo de curativo	Gaze e fita microporosa	6	12	18	0,470*	1,3 (0,612; 2,813)
	Transparente impermeável	14	18	32		
Volume da solução	≤500 ml	4	10	14	0,353^†^	0,6 (0,260; 1,589)
	1.000 ml	16	20	36		
Calibre do cateter	18G	7	14	21	0,413*	1,3 (0,650; 2,783)
	20G; 22G	13	16	29		

*Qui-quadrado de Pearson; ^†^Teste Exato de Fisher

Quando se avalia a relação do desfecho com idade, tempo de uso do dispositivo e pressão utilizada no sistema de bolsa pressurizadora evidencia-se que a pressão da bolsa teve associação com a retirada do cateter antes do indicado (p=0,046), sendo que as pressões abaixo do adequado estão relacionadas com as perdas e retiradas antes do previsto (Tabela 2). Além disso, a magnitude dessa diferença pode ser considerada de média intensidade pelo valor de g de Hedges.

## Discussão

Este estudo elencou como principais complicações a retirada precoce do dispositivo por obstrução (36,54%) e flebite (52,00%). A literatura descreve como complicações da PAI dor local, parestesia, hematoma, sangramento, complicações isquêmicas, embolia, trombose vascular e oclusão, lesão do vaso, síndrome compartimental, formação de pseudoaneurisma, abscesso, infecção, sepse e lesão nervosa local^(1-4,9)^. Dentre essas, destacam-se, como mais comuns, oclusão do vaso, sangramento no sítio de inserção e formação de hematomas. Contudo, embora mais frequentes, essas complicações podem ser identificadas e tratadas de forma eficaz, o que reduz a possibilidade de complicações maiores^(4)^.

Outra complicação descrita na literatura refere-se ao uso da monitorização invasiva da pressão arterial como fator que aumentou as chances de lesão miocárdica em comparação com pacientes monitorados por meio da pressão arterial não invasiva^(17)^.

Revisão da literatura avaliou 19.617 punções na artéria radial e descreveu a oclusão temporária da artéria (incidência de 19,70%) como a principal complicação desse sítio de punção. Ainda nessa revisão, a segunda artéria mais utilizada para monitoramento hemodinâmico foi a femoral, sendo que, nesse local, a oclusão temporária foi relatada em apenas 1,18% dos casos. Menores índices desta complicação na artéria femoral podem ser atribuídos ao maior diâmetro deste vaso. Outras complicações descritas por esses autores, como dano isquêmico, sepse e formação de pseudoaneurisma, foram semelhantes para as artérias radial, femoral e axilar^(2)^.

Identificou-se, ainda, que a maior parte das punções foi realizada na artéria radial (86%). Este dado corrobora o encontrado em revisão de literatura, a qual mencionou a artéria radial como local mais utilizado para a instalação do dispositivo de monitorização da pressão arterial invasiva. Segundo os autores, isso ocorre em decorrência da baixa taxa de complicação e do fácil acesso deste sítio^(2)^.

Neste estudo, o fator que demonstrou relação com a retirada precoce do dispositivo foi a pressão da bolsa (p=0,046), sendo que os valores menores estavam relacionados com maior número de perda do dispositivo. Recomenda-se que os valores pressóricos estejam próximos a 300 mmHg para manutenção da permeabilidade adequada do dispositivo^(3,18)^. No presente estudo, o valor médio encontrado nos casos com retirada do dispositivo antes do recomendado foi de 262,22 mmHg.

Sabe-se que a manutenção de níveis pressóricos adequados no dispositivo de PAI é um cuidado essencial para a permeabilidade do acesso, sendo que a pressão exercida objetiva evitar o refluxo de sangue e a obstrução do cateter. Nesse sentido, algumas referências sinalizam o uso de heparina como método preventivo para a formação de trombos^(19)^. No presente estudo, não houve a utilização de heparina na solução fisiológica em nenhum dispositivo.

Outro dado encontrado na literatura aponta que a anticoagulação pré ou pós-procedimento, bem como o uso de vasodilatadores e compressão local, reduzem o risco de oclusão arterial após a punção arterial^(9)^. No entanto, neste estudo não foram encontradas relações entre o uso de anticoagulantes sistêmicos (p=0,745) e os desfechos de complicação, tal como observado em estudos randomizados e de revisão sistemática já publicados^(9,20-21)^.

Estudo conduzido com pacientes com quadros de sepse objetivou avaliar a eventual diferença nas medidas de pressão invasiva e não invasiva em pacientes com choque séptico e identificou associação do uso de vasopressores com probabilidades aumentadas de uma discrepância de pressão arterial clinicamente significativa entre as medidas invasiva e não invasiva. Uma pontuação mais elevada no escore SOFA e níveis mais elevados de lactato sérico também foram associados a uma maior probabilidade de uma discrepância da pressão arterial, levando às alterações clínicas no tratamento^(22)^. No presente estudo, havia a intenção de avaliar a relação entre o uso de drogas vasoativas e as complicações; entretanto, pelo limitado número de participantes que não utilizaram esses medicamentos, essa análise não foi realizada.

Ainda no presente estudo, a complicação mais prevalente foi a flebite, responsável pela retirada precoce do dispositivo em 52% dos casos. Uma coorte que avaliou e documentou casos de flebites em acessos venosos encontrou a incidência dessa complicação em 43,2% dos casos e apontou, como fatores relacionados, o tempo de internação e o número de cateteres inseridos^(23)^. Já estudo de coorte multicêntrico avaliou 3429 cateteres periféricos em pacientes críticos, tendo constatado os seguintes principais fatores de risco para o desenvolvimento de flebite: institucionais, inerentes do próprio paciente, tipo de cateter e fatores induzidos por medicamentos^(24)^. Destaca-se que esses estudos avaliaram flebite em acesso venoso, sendo escassos estudos para avaliar essa relação em cateteres arteriais.

Sobre a flebite em acessos arteriais, estudo de revisão^(9)^ apontou que o cateterismo arterial apresenta baixo risco de infecção, com incidência menor que 1%. Ademais, descreveu que infecções em acessos arteriais podem estar relacionadas ao desenvolvimento de pseudoaneurisma no vaso puncionado, tal como relatado em outra revisão, segundo a qual infecções neste tipo de acesso são raras^(25)^.

A redução da incidência de infecções em acessos arteriais pode ocorrer mediante a implementação de alguns fatores, como técnica de inserção asséptica e adequada desinfecção do local de inserção. Entretanto, a antibioticoterapia profilática não parece reduzir o risco para infecções relacionadas ao cateter^(2)^.

Um dos fatores relacionados ao maior risco para complicações infecciosas e sepse é a permanência do cateter arterial, especialmente se a punção permanecer por mais de 96 horas^(2)^. Neste estudo, a média de tempo de permanência do cateter foi de 4,36 dias (dp: 3,504), com mínimo de 1 dia e máximo de 16.

O material do cateter também é descrito como fator a ser considerado, uma vez que alguns materiais parecem apresentar maior resistência aos determinados micro-organismos^(2)^. No estudo em tela, contudo, este dado não pôde ser avaliado, pois foi utilizado nas punções um único tipo de cateter.

O estudo teve como limitações o fato de ter sido desenvolvido em um único hospital, o que impossibilitou a comparação com outros protocolos de uso da pressão arterial invasiva (PAI), como outros cateteres, diferentes tipos de curativos, formas de fixação, técnica para punção ou sítios de punção mais diversos. Acresce-se que a amostra por conveniência permite a presença de vieses na análise.

Havia interesse em verificar se o uso de drogas vasoativas teria efeito na incidência de complicações, entretanto, dada a baixa frequência de participantes que não utilizaram esses medicamentos, não foi possível obter um resultado confiável neste quesito. Essa também pode ser descrita como uma limitação do estudo. Sugere-se que essa variável seja novamente avaliada em pesquisas futuras, com maiores amostras.

O estudo traz como contribuições a importância da adequada manutenção da pressão na bolsa do dispositivo de monitorização. Trata-se de um cuidado de enfermagem simples de ser avaliado e executado, capaz de reduzir as complicações. Ademais, como as flebites foram as principais complicações encontradas, destaca-se a necessidade de cuidados rigorosos para a prevenção de infecções associadas ao sítio de punção.

## Conclusão

O fator relacionado às complicações do sistema de monitorização da PAI encontrado no presente estudo foi a pressão da bolsa, que teve associação com a retirada do cateter antes do momento indicado. As principais complicações associadas com a retirada do cateter antes do indicado foram a flebite e a obstrução.
